# BRMDA: prediction model for potential microbe-drug associations based on bilinear attention networks and random forest

**DOI:** 10.3389/fgene.2026.1757318

**Published:** 2026-03-26

**Authors:** Ge Yu, Fang Chen, Hui Chen, Shichang Tang, Mingmin Liang, Xianzhi Liu, Bin Zeng, Lei Wang

**Affiliations:** 1 School of Intelligent Equipment, Hunan vocational College of Electronic and Technology, Changsha, China; 2 School of Continuing Education, Central South University of Forestry and Technology, Changsha, China; 3 School of Information Engineering, Hunan vocational College of Electronic and Technology, Changsha, China; 4 Big Data Innovation and Entrepreneurship Education Center of Hunan Province, Changsha University, Changsha, China

**Keywords:** bilinear attention networks, heterogeneous graph, microbe-drug association prediction, multimodal feature fusion, random forest

## Abstract

**Introduction:**

Uncharted microbe-drug relationships constitute an under-exploited reservoir of therapeutic leads. In this manuscript, we introduced a hybrid framework named BRMDA by coupling a bilinear attention network with a random-forest classifier to systematically expose latent microbe-drug associations.

**Methods:**

Firstly, BRMDA integrated multiple drug-centric, microbe-centric, and disease-centric similarity profiles, along with experimentally validated microbe–drug associations, to construct a unified heterogeneous graph. And then, the bilinear attention network and random-forest classifier were employed to compute the predicted scores for potential microbe-drug associations based on the newly constructed unified heterogeneous graph. Next, benchmarking experiments were conducted under a rigorous five-fold cross-validation protocol using the MDAD dataset to validate the prediction performance of BRMDA. Additionally, case studies were further performed, focusing on front-line antibiotics including amoxicillin and ciprofloxacin as well as clinically relevant pathogens including *Bacillus* cereus and *Mycobacterium tuberculosis*, to evaluate the translational validity of the proposed model.

**Conclusion:**

Intensive experimental results demonstrated that BRMDA outperformed seven state-of-the-art competitors in terms of both AUC and AUPR, and 9 out of the top 10 associations predicted by the model were corroborated by independent literature evidence. These findings underscored the accuracy and translational potential of BRMDA, offering a data-driven compass for antimicrobial discovery and microbe-oriented therapeutic design.

## Introduction

Microbes are an extensive and enigmatic group in the biosphere. They are so tiny that they are usually invisible to the naked eye and can only be observed with the aid of microscopes (Huttenhower et al., 2012). There is a vast diversity of microbes, including bacteria, fungi, and viruses (Liang et al., 2020). Microbes are present throughout the human body (Gill et al., 2006). They not only aid in food absorption and gut health but also enhance mucosal and systemic immunity by balancing the gut microbiota (Ventura, 2009; Sommer and Bckhed, 2013). In the gut, these microbes depend on each other and benefit mutually. When the balance of gut microbiota is disrupted, it may lead to various diseases (Liang et al., 2024). Moreover, many studies have shown that microbes and drugs interact significantly during treatment (Mc et al., 2021). Therefore, understanding the relationship between microbes and drugs is crucial for effective disease treatment.

In-depth biological research has revealed the key connections between drugs and microbes. However, the huge consumption of human resources, materials and time in biological experiments has become a bottleneck restricting the in-depth development of research. In recent years, with the rapid development of research tools, computational methods have been increasingly applied. These methods are specifically designed to predict the interactions between drugs and microbes (Lei et al., 2022). At the same time, researchers have also constructed experimentally validated databases of microbe-drug associations. Databases such as MDAD (Sun et al., 2018) provide valuable data support for relevant research. Based on these foundations, Lai et al. (2025) introduces a novel prediction model called GTFKAN, which leverages the integration of Graph Transformer and Fourier Kolmogorov-Arnold Networks to forecast the interactions between microbes and drugs. Yang et al. (2025) developed a predictive model named NNSFMDA for microbial-drug associations, which integrates Lightweight Transformer Model with Bounded Nuclear Norm Minimization. Liang J. et al. (2025) designed a model named GRL-PUL, which is based on graph representation learning and forward label-free learning, to predict the associations between microbes and drugs. Li et al. (2025) proposed a multi similarity ensemble method with pre-completion and error correction functions for predicting the association between microbes and drugs. Based on the transformer principle, Xiao et al. (2025). Designed a comparative learning model for microbe-drug association prediction graphs.

**FIGURE 1:**
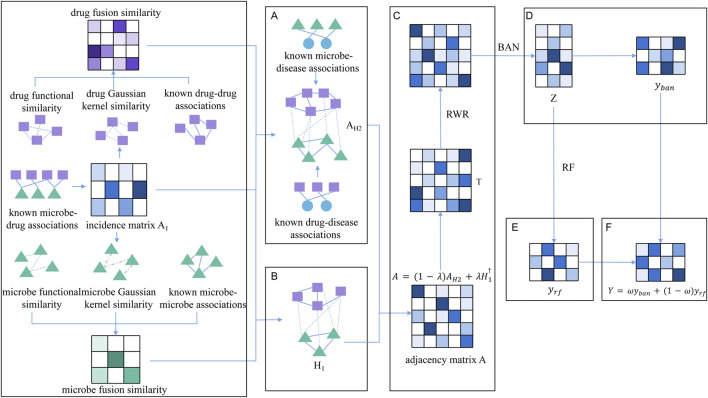
The overall structure diagram of BRMDA. **(A)** The heterogeneous network consisting of microbes, drugs, and diseases was established by integrating microbe similarity networks, drug similarity networks, and the known associations among microbes, drugs and diseases. **(B)** The heterogeneous microbe-drug network was constructed by integrating microbe similarity networks, drug similarity networks, and the known microbe-drug associations. **(C)** These two heterogeneous networks were fused, and node embeddings were generated via the random walk with restart. **(D)** Predicting potential microbe-drug associations by BAN. **(E)** Predicting potential microbe-drug associations by RF. **(F)** Predicting the final scores of potential microbe-drug associations.

Inspired by Liang M. et al. (2025) and Kuang et al. (2024a), we have developed a novel prediction model, BRMDA, that integrates the concepts of bilinear attention networks and random forest algorithms to predict the correlation between microbes and drugs. As illustrated in Figure 1, the key contributions of BRMDA are highlighted by the following innovative aspects:

1. By integrating microbe similarity networks, drug similarity networks, and the known relationships among microbes, drugs, and diseases, we constructed a novel, comprehensive heterogeneous microbe-drug network, designated as Network A .
2. To obtain more reliable scores for latent microbe-drug associations, we first performed a random walk on A to generate node embeddings, which would then be fed into a BAN module that explicitly models cross-modal structural relationships, yielding an intermediate fused representation. Next, this representation would be simultaneously forwarded to a fully connected layer and a Random-Forest classifier, producing two independent probability predictions. The final association scores were obtained by a weighted average of the two outputs, balancing the strengths of deep learning and ensemble learning.

## Materials and methods

### Data sources

To evaluate the predictive capabilities of the BRMDA model, we utilized the MDAD dataset. This dataset, meticulously assembled by Sun et al. (2018) in 2018, represents a comprehensive collection of microbe-drug associations. It was derived from multiple authoritative drug databases, including TTD and DrugBank, as well as extensive scholarly literature. After eliminating duplicate entries, the dataset comprises 1,373 distinct drugs and 173 unique microbes, interconnected by 2,470 associations. Subsequently, we gathered known drug-disease associations and known microbe-disease associations from the dataset introduced by Wang et al. (Lei et al., 2022) in 2022. In the experimental process, only diseases that were associated with at least one drug and one microbe in the MDAD dataset, along with the associations related to these diseases, were retained. Consequently, we ultimately identified 109 diseases, 73 microbes, 233 drugs, 1,121 specific drug-disease associations, and 402 particular microbe-disease associations. In addition, we have collected 138 known microbe-microbe interactions, involving 123 microbes in MDAD, and 5,586 known drug-drug relationships from the dataset compiled by Deng et al. (2022), which includes 1,228 drugs in MDAD. Table 1 presents detailed statistical information.

**TABLE 1:** The specific statistical data for MDAD dataset.

Type	Microbes	Drugs	Disease	Associations
Microbe-drug associations	173	1,373	-	2,470
Microbe-disease associations	73	-	109	402
Drug-disease associations	-	233	109	1,121
Drug-drug associations	-	1,228	-	5,586
Microbe-microbe associations	123	-	-	138

#### Negative sampling and cross-validation

As MDAD provides curated positive associations but no confirmed negatives, we constructed pseudo-negative instances by uniformly sampling from unlabeled microbe–drug pairs. For evaluation, we used a balanced setting by sampling the same number of pseudo-negatives as positives (1:1). Specifically, we do not duplicate (oversample) positive pairs; instead, all positives are retained, and negatives are randomly sampled from the zero (unknown) entries (i.e., negative down-sampling). To reflect increasing class imbalance, we additionally evaluate multiple negative-to-positive ratios in the supplementary experiments. We then performed stratified five-fold cross-validation on the resulting labeled pairs.

For each fold, the threshold for ACC/F1 is determined on the training split by maximizing the F1-score on the training PR curve, and then applied to the corresponding test split to binarize predictions and compute ACC/F1.

In addition to the balanced setting (1:1), we further evaluated robustness under different pseudo-negative sampling ratios (Neg:Pos = 3:1, 5:1, 10:1). In each fold, pseudo-negative pairs were randomly sampled from unobserved microbe–drug pairs with the specified ratio, while the cross-validation splitting protocol remained unchanged. The corresponding results are reported in Table 2.

**TABLE 2:** Performance under different pseudo-negative sampling ratios (Neg:Pos).

Neg:Pos	AUC	AUPR	F1	ACC
1:1	0.9857	0.9792	0.9569	0.9569
3:1	0.9881	0.9618	0.9273	0.9634
5:1	0.9895	0.9497	0.9024	0.9673
10:1	0.9904	0.9232	0.8765	0.9772

As the negative ratio increases, AUC remains stable (0.9857–0.9904), indicating consistent ranking capability. Meanwhile, AUPR and F1 decrease with increasing imbalance (AUPR: 0.9792 → 0.9232; F1: 0.9569 → 0.8765), which is expected due to the sensitivity of PR-based and threshold-dependent metrics to class prevalence. ACC increases (0.9569 → 0.9772) because the majority class becomes negative. These results suggest that our method is robust to different pseudo-negative sampling ratios.

To prevent information leakage during feature construction, positive edges in the test fold were removed from the training microbe-drug adjacency before computing similarity and random-walk-based features.

## Methods

### Microbe-drug incidence matrix

Leveraging the correlation data provided, we initially established an association matrix $A_{1}$ between microbes and drugs. In this matrix, each entry is designated as 1 if a relationship exists between a specific drug $d_{i}$ and a specific microorganism $m_{j}$ ; otherwise, it is assigned a value of 0. The specific formula is presented in Equation 1.

$$A_{1}\left(i,j\right)=\left\{\begin{array}{l} 1,if\;d_{i}\;associats\;with\;m_{j}\\ 0,otherwise \end{array}\right.$$ (1)

### Microbe/drug Gaussian kernel similarity

The Gaussian kernel similarity, based on the Gaussian kernel function, is a popular method in microbe-drug association prediction. It assumes similar microbes have similar interactions with the same drug. The Gaussian kernel similarity between drugs $d_{i}$ and $d_{j}$ can be computed via Equation 2.

$$D_{GIP}=\exp\left(‐{\gamma_{d}\left\|R\left(d_{i}\right)‐R\left(d_{j}\right)\right\|}^{2}\right)$$ (2)

In the Gaussian kernel similarity, $\left\|R\left(d_{i}\right)‐R\left(d_{j}\right)\right\|$ denotes the Euclidean distance between two diseases, while the parameter $\gamma_{d}$ , as shown in Equation 3, plays a crucial role in determining how this distance impacts the similarity measurement.

$$\gamma_{d}=1/\left(\frac{1}{n_{d}}\sum_{i=1}^{n_{d}}{\left\|R\left(d_{i}\right)\right\|}^{2}\right)$$ (3)

The Gaussian kernel similarity $M_{GIP}$ can also be used to assess the similarity between microbes. In Equations 2–5, $R\left(d_{i}\right)$ and $R\left(m_{i}\right)$ denote the interaction profiles of drug $d_{i}$ and microbe $m_{i}$ , respectively, which are defined as the corresponding column and row vectors of the microbe–drug incidence matrix (A). Each entry of these vectors is binary, indicating the presence or absence of a known microbe–drug association.

$$M_{GIP}=\exp\left(‐{\gamma_{m}\left\|R\left(m_{i}\right)‐R\left(m_{j}\right)\right\|}^{2}\right)$$ (4)

$$\gamma_{m}=1/\left(\frac{1}{n_{m}}\sum_{i=1}^{n_{m}}{\left\|R\left(m_{i}\right)\right\|}^{2}\right)$$ (5)

### Microbe/drug functional similarity

Microbe functional similarity is calculated using the Kamneva (2017), based on microbial gene family analysis. It starts with building a microbial protein-protein functional association network using the STRING dataset (Szklarczyk et al., 2019). In this network, nodes are gene families, and edges represent genetic neighborhood scores. The Kamneva tool then creates matrix $M_{FUN}$ by comparing the edge scores between two microbes to the total link scores of their gene families.

Moreover, SIMCOMP (Hattori et al., 2010) uses drug chemical structures and molecular formulas to measure structural similarity. It matches nodes and edges in chemical diagrams via algorithms to identify the largest common substructure, thereby calculating similarities between drug frameworks. This approach enables the construction of a drug functional similarity matrix $D_{FUN}$ .

### Microbe/drug fusion similarity

Undoubtedly, it can be challenging to compare all microbes using Gaussian kernel similarity or functional similarity alone. To overcome this hurdle, we have developed a novel similarity matrix by integrating known association data between microbes with two distinct types of similarity information, as detailed in Equation 6.

$$S_{m}^{v}\left(m_{i},m_{j}\right)=\left\{\begin{array}{l} 1,if\;there\;is\;a\;known\;association\;between\;m_{i}\;and\;m_{j}\\ \left(M_{GIP}+M_{FUN}\right)/2,otherwise \end{array}\right.$$ (6)

Likewise, the drug fusion matrix can be derived from Equation 7.

$$S_{d}^{v}\left(d_{i},d_{j}\right)=\left\{\begin{array}{l} 1,if\;there\;is\;a\;known\;association\;between\;d_{i}\;and\;d_{j}\\ \left(D_{GIP}+D_{FUN}\right)/2,otherwise \end{array}\right.$$ (7)

#### Constructing the heterogeneous network $H_{1}$

We integrated the microbe-drug incidence matrix with the drug fusion similarity matrix and the microbe fusion similarity matrix to construct a unified matrix, as detailed in Equation 8.

$$H_{1}=\left[\begin{array}{cc} S_{m}^{v} & A_{1}\\ A_{1}^{T} & S_{d}^{v} \end{array}\right]\in R^{\left(n_{r}+n_{m}\right)\times \left(n_{r}+n_{m}\right)}$$ (8)

### Feature construction

#### Constructing the structure-enhanced heterogeneous graph $A_{H2}$

Matrix $A_{H2}$ serves as a comprehensive adjacency matrix designed to capture the relationships among three types of nodes in a heterogeneous graph: microbes, diseases, and drugs. Its structure is detailed in Equation 9.

$$A_{H2}=\left[\begin{array}{ccc} {\lambda_{m}S}_{m}^{v} & A_{mdis} & 0\\ A_{mdis}^{⊤} & 0 & A_{ddis}\\ 0 & A_{ddis}^{⊤} & {\lambda_{d}S}_{d}^{v} \end{array}\right]$$ (9)

As shown in Equation 9, the diagonal blocks of $A_{H2}$ describe within-type similarities, where $S_{m}^{v}$ and $S_{d}^{v}$ denote the microbe fusion similarity matrix and the drug fusion similarity matrix, respectively. The off-diagonal blocks describe cross-type associations, where $A_{mdis}$ represents the known microbe–disease associations and $A_{ddis}$ represents the known drug–disease associations; their transposed blocks are used to keep the adjacency structure symmetric. The remaining blocks are set to 0, indicating that no disease–disease similarity is introduced and no direct microbe–drug edges are defined in $A_{H2}$ . Notably, the direct microbe–drug incidence information is constructed in Equation 8, and is then extended via zero-padding and embedded into the node space of $A_{H2}$ for the weighted fusion in Equation 10.

Among them, $\lambda_{m}$ and $\lambda_{d}$ are adjustable coefficients used to control the importance of similarity edges.

#### Constructing the unified adjacency matrix A

Extend matrix $H_{1}$ to matrix $H_{1}^{↑}$ via zero-padding, embed it into the node space of matrix $A_{H2}$ , and then perform weighted fusion to derive a unified adjacency matrix, as shown in Formula 10.

$$A=\left(1‐\lambda \right)A_{H2}+\lambda \cdot H_{1}^{↑}$$ (10)

#### Constructing the node feature matrix

Based on the unified adjacency matrix A , construct the transition probability matrix T as depicted in Equation 11.

$$T=D^{‐1}A$$ (11)

Where $D_{ii}=\sum_{j}A_{ij}$ .

To more efficiently capture both the local and global topological intrinsic properties of the nodes, we further implemented an enhanced Random Walk with Restart (RWR) mechanism on T , as detailed in Equation 12.

$$r_{i}^{t+1}=\alpha T^{T}r_{i}^{t}+\left(1‐\alpha \right)\varepsilon_{i}$$ (12)

In Equation 12, α denotes the restart probability. $T^{T}$ is the transpose of T , and $\varepsilon_{i}$ represents the initial probability vector for node i , which is defined in Equation 13.

$$\varepsilon_{ij}=\left\{\begin{array}{l} 1,if\;i=j\\ 0,otherwise \end{array}\right.$$ (13)

The RWR will be conducted for each node as the starting point to derive its structural steady-state distribution vector $r_{i}^{*}$ . The concatenated results for all nodes can be utilized to separately construct the microbe embedding matrix $R_{m}\in R^{n_{m}\times d}$ , drug embedding matrix $R_{d}\in R^{n_{d}\times d}$ , and disease embedding matrix $R_{dis}\in R^{n_{dis}\times d}$ . Where $R_{m}$ represents the distribution of the spreading influence of each microorganism in the heterogeneous graph, $R_{d}$ is the structural representation of drug nodes, and $R_{dis}$ is a disease node acting as an intermediary, and its structural embedding reflects bridging ability.

### Multimodal feature fusion and BAN training

Bilinear Attention Networks (BAN) is a deep learning model designed for multimodal fusion, with its core principle being the precise capture of interactions between image and text features via bilinear operations (Liu et al., 2025). This network employs two key technologies to strengthen feature interactions and manage complex data relationships: bilinear transformation and attention mechanisms. Specifically, bilinear transformation processes input features using a weight matrix and an additive bias, enabling the precise extraction of nuanced correlations within complex datasets. We apply bilinear transformation to map the three embedding matrices $R_{m}$ , $R_{d}$ and $R_{dis}$ into a unified semantic space, as illustrated in Equation 14. Through this transformation, the output results ${\tilde{r}}_{m}$ , ${\tilde{r}}_{d}$ , and ${\tilde{r}}_{dis}$ for microbes, drugs, and diseases are obtained.

$$\begin{array}{rr} {\tilde{r}}_{m} & =W_{m}R_{m}+b_{m}{\tilde{r}}_{d}=W_{d}R_{d}+b_{d}{\tilde{r}}_{dis}=W_{dis}R_{dis}+b_{dis} \end{array}$$ (14)

Among them, $W_{m}$ , $W_{d}$ , and $W_{dis}$ denote the transformation matrices for microbes, drugs, and diseases, respectively, while $b_{m}$ , $b_{d}$ , and $b_{dis}$ represent the corresponding bias terms for microbes, drugs, and diseases. Next, employ a three-way bilinear interaction to capture the structural relationships between different modalities, as illustrated in Equation 15.

$$\begin{array}{r} z_{md}=ReLU\left({\tilde{r}}_{m}^{T}W_{md}{\tilde{r}}_{d}\right)z_{mdise}=ReLU\left({\tilde{r}}_{m}^{T}W_{mdise}{\tilde{r}}_{dis}\right)z_{ddise}=ReLU\left({\tilde{r}}_{d}^{T}W_{ddise}{\tilde{r}}_{dis}\right) \end{array}$$ (15)

In Equation 15, $z_{md}$ is the interaction between microbes and drugs, $z_{mdise}$ is the interaction between microbes and diseases, and $z_{ddise}$ is the interaction between drugs and diseases. ${\tilde{r}}_{m}^{T}$ , ${\tilde{r}}_{d}^{T}$ , and ${\tilde{r}}_{dis}^{T}$ are the transpose matrices of ${\tilde{r}}_{m}$ , ${\tilde{r}}_{d}$ , and ${\tilde{r}}_{dis}$ , respectively, while $W_{md}$ , $W_{mdise}$ and $W_{ddise}$ represent the weight matrices from the input layer to the hidden layer for microbe-drug, microbe-disease, and drug-disease, respectively. Construct an intermediate representation following feature fusion, as depicted in Equation 16.

$$Z=\left[\begin{array}{c} z_{md}\\ z_{mdise}\\ z_{ddise} \end{array}\right]$$ (16)

Then send Z to the fully connected layer to output the classification result, as depicted in Equation 17.

$$h=ReLU\left(W_{h}z_{i}+b_{h}\right)$$ (17)

$$y_{ban}=Softmax\left(W_{out}h+b_{out}\right)$$ (18)

In Equation 17, $W_{h}$ is the weight matrix from the input layer to the hidden layer, $b_{h}$ is the bias vector of the hidden layer, and $z_{i}$ represents the input vector in matrix Z . In Equation 18, $W_{out}$ and $b_{out}$ represent the weight matrix and bias vector from the hidden layer to the output layer, respectively. $y_{ban}$ represents the direct prediction result of the BAN model, reflecting the end-to-end multimodal inference capability of the model.

### Random forest classification prediction model

Random Forest (RF) is a powerful ensemble learning algorithm that has gained widespread application in both classification and regression tasks (Kuang et al., 2024b). By constructing an array of decision trees and amalgamating their predictions, it significantly enhances the model’s accuracy and stability.

Step 1: Feed $z_{i}$ , the intermediate fusion feature of BAN, into the RF classifier.
Step 2: Build the training set $D=\left\{\left(z_{i},y_{i}\right)\right\}$ . Among them, $y_{i}$ denotes the binary label assigned to each known microbe-drug association.
Step 3: Harnessing the RF model, we derive the final prediction matrix $y_{rf}=RF\left(z_{i}\right)$ .

### Final predicted microbe-drug association scores

$y_{ban}$ captures the raw, end-to-end multimodal inference of the BAN model, while $y_{rf}$ distills the same representation through a conventional classifier, trading complexity for added robustness and broader generalization. The two outputs are ultimately fused via a weighted average (Equation 19) to yield the final prediction score.

$$Y=\omega y_{ban}+\left(1-\omega \right)y_{rf}$$ (19)

Here, ω is swept from 0 to 1 in steps of 0.005 and for every split of the 5-fold CV the model’s scores are collected and the AUC is computed then the weight that yields the highest mean AUC is retained as optimal.

## Experiments and results

In this section, we first calibrated the model by systematically perturbing its core parameters. Subsequently, six state-of-the-art approaches were benchmarked against BRMDA. To further corroborate the findings, two exemplar microbe-drug pairs were independently examined.

### Parameter sensitivity analysis

**FIGURE 2:**
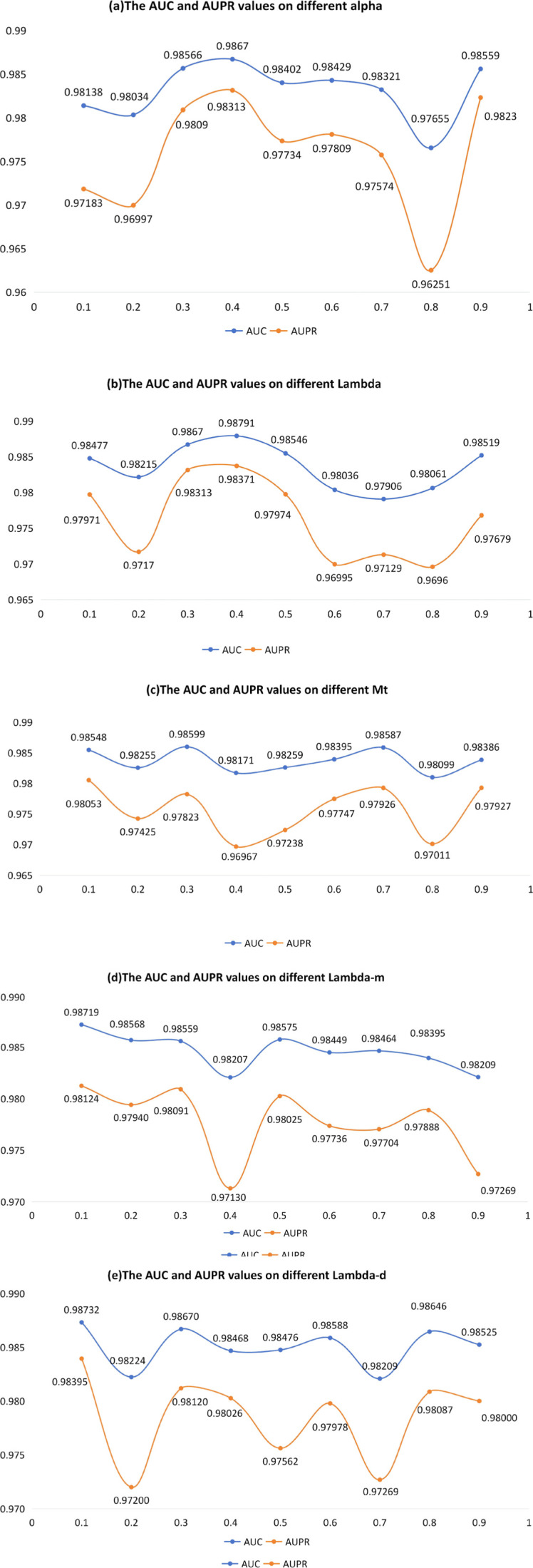
The AUC and AUPR values on different parameter sensitivity analysis. **(a)** The AUC and AUPR values on different alpha. **(b)** The AUC and AUPR values on different lambda. **(c)** The AUC and AUPR values on different Mt. **(d)** The AUC and AUPR values on different lambda-m. **(e)** The AUC and AUPR values on different lambda-e.

We first determined the final hyperparameter configuration via a joint grid search. Then, for interpretability, we report one-factor-at-a-time sensitivity curves in Figure 2 by varying a single hyperparameter while fixing the others to the selected optimal configuration.

Five parameters that critically govern model prediction were identified and analyzed in the context of the real scenario. The first, α in Equation 12, originates from set $\left\{0.1,0.2,0.3,0.4,0.5,0.6,0.7,0.8,0.9\right\}$ ; the second, λ in Equation 10, is taken from set $\left\{0.1,0.2,0.3,0.4,0.5,0.6,0.7,0.8,0.9\right\}$ ; the third, Mt is the feature considered for each split in a random forest, following standard random-forest practice, its candidate values are drawn from set $\left\{0.094,0.125,0.3,0.5,0.7\right\}$ . Moreover, the fourth, $\lambda_{m}$ in Equation 9, and the fifth, $\lambda_{d}$ in Equation 9, are adjustable coefficients used to control the importance of similarity edges, and their candidate values are drawn from set $\left\{0.1,0.2,0.3,0.4,0.5,0.6,0.7,0.8,0.9\right\}$ . Thereafter, model performance under the selected hyper-parameter set was quantified by five-fold cross-validation, with the area under the receiver-operating-characteristic curve (AUC) and the area under the precision-recall curve (AUPR) serving as the primary metrics. The outcomes are displayed in Figure 2: panel (a) illustrates the results obtained under varying values of α , panel (b) those under different λ settings, panel (c) the corresponding findings for diverse Mt levels. Panel (d) the corresponding findings for diverse $\lambda_{m}$ levels, and panel (e) the corresponding findings for diverse $\lambda_{d}$ levels. As shown in Figure 2, optimal model performance is achieved with the following parameter settings: $\alpha =0.4,\lambda =0.4,Mt=0.125,\lambda_{m}=0.1,\lambda_{d}=0.8$ .

The sensitivity curves in Figure 2 exhibit a non-monotonic pattern, suggesting that the involved hyperparameters control a balance between local neighborhood specificity and global structural propagation in the heterogeneous network. Extreme settings tend to over-emphasize either overly local transitions or overly diffuse propagation, which can weaken the discriminative ranking of candidate associations. Therefore, we select the default configuration from a stable region where performance is consistently high, aligning with the intended design of combining structure-enhanced diffusion with interaction modeling.

## Comparison with advanced methods

To rigorously benchmark BRMDA, we compared it with seven leading algorithms. Fairness was guaranteed by retaining each competitor’s original hyper-parameters and subjecting every model BRMDA included to the same five-fold cross-validation split on the MDAD dataset.

- HMDAKATZ (Zhu et al., 2019): Built upon the KATZ metric, the approach quantifies proximity within the heterogeneous graph to infer putative microbe-drug associations.
- SCSMDA (Tian et al., 2023): This approach integrates structure-enhanced contrastive learning with self-paced negative sampling to predict microbe-drug associations.
- GSAMDA (Tan et al., 2022): This model utilizes graph attention networks and sparse autoencoders to provide a new approach for predicting potential microbial drug interactions.
- HMDA-Pred (Fan et al., 2020): This model predicts microbe-drug associations by integrating multi-omics data through a network-consistency projection framework.
- MDASAE (Fan et al., 2023): This model leverages a stacked autoencoder and a multi-head attention mechanism to jointly mine and interpret the intricate interplay between microbes and drugs.
- MHBVDA (Cheng et al., 2022): This model predicts potential virus-drug associations by reconstructing a virus-drug heterogeneous network via matrix decomposition, heterogeneous graph inference, and bounded nuclear-norm regularization.
- NIRBMMDA (Qu et al., 2023): This model infers potential microbe-drug associations by ensemble-integrating neighborhood-based inference (NI) and a restricted Boltzmann machine (RBM) using known associations and integrated similarity information.

**FIGURE 3:**
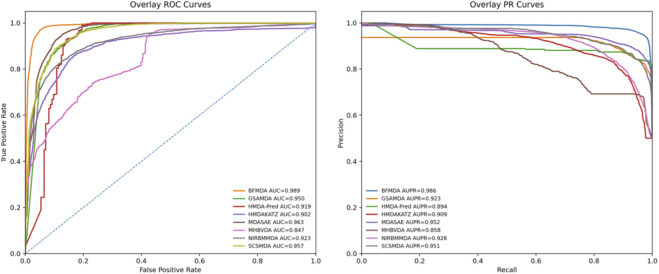
AUC and AUPR curves of seven competitive methods based on the MDAD dataset.

We benchmarked all methods under their default settings in a rigorous 5-fold cross-validation on the MDAD dataset, recording AUC and AUPR. As Table 3 and Figure 3 show, BRMDA outperforms every comparator, setting a new state-of-the-art for microbe–drug association prediction.

**TABLE 3:** The results of seven comparison methods.

Methods	AUC	AUPR
HMDAKATZ	0.9012 ± 0.0013	0.9089 ± 0.0071
SCSMDA	0.9566 ± 0.0037	0.9498 ± 0.0059
GSAMDA	0.9472 ± 0.0017	0.9318 ± 0.0011
HMDA-pred	0.9183 ± 0.0015	0.8953 ± 0.0311
MDASAE	0.9613 ± 0.0021	0.9518 ± 0.0011
MHBVDA	0.8746 ± 0.0025	0.8583 ± 0.0011
NIRBMMDA	0.9233 ± 0.0027	0.9287 ± 0.0036
BRMDA	0.9887 ± 0.0025	0.9858 ± 0.0011

Beyond reporting the mean scores in Table 3, we interpret the improvements from two complementary perspectives. First, the proposed method consistently achieves higher discrimination and ranking quality across the reported metrics, indicating that the learned representations capture more informative cross-entity signals. Second, the improvement in AUPR is particularly meaningful for microbe–drug association prediction, where positive associations are typically sparse; thus, AUPR better reflects the quality of prioritizing high-confidence candidates for downstream validation. The overall trend in Figure 3 further supports the consistency of this advantage across the reported settings.

## Case study

To rigorously evaluate the predictive performance of the BRMDA model, this study conducted targeted case studies using two clinically widely-used drugs, amoxicillin and ciprofloxacin, and two representative microbes, *Bacillus* cereus and *Mycobacterium tuberculosis*.

Amoxicillin (Huttner et al., 2020), a representative β-lactam antibiotic of the penicillin class, has attracted sustained attention in the field. Numerous investigations have demonstrated potent and reproducible activity against *Bacillus subtilis* (Kovai et al., 2023), *Clostridium perfringens* (Elbadawy and Aboubakr, 2017), and *Listeria* monocytogenes (Grayo et al., 2008). Microbes potentially interacting with amoxicillin were first ranked by BRMDA-predicted scores. After excluding the three drug-microbe pairs already recorded in the MDAD dataset, the ten highest-scoring organisms were selected for targeted validation. As shown in Table 4, nine of these ten predicted associations are supported by independent literature indexed in PubMed.

**TABLE 4:** The top 10 amoxicillin associated candidate microbes on MDAD.

Microbe	Evidence
Firmicutes	PMID: 39215861
*Pseudomonas aeruginosa*	PMID: 31026042
Gardnerella vaginalis	PMID: 35690718
*Staphylococcus* capitis	PMID: 35999775
*Bacteroides* eggerthii	NA
Eikenella corrodens	PMID: 11600394
*Streptococcus* salivarius	PMID: 27569711
*Enterococcus* faecium	PMID: 28670644
*Staphylococcus aureus*	PMID: 31396174
Eggerthella lenta	PMID: 25520446

Ciprofloxacin (Terp and Rybak, 1988), a second-generation fluoroquinolone, exhibits rapid bactericidal activity and an exceptionally broad antimicrobial spectrum. Clinically, it is a first-line option for acute and chronic urinary-tract infections as well as for life-threatening systemic infections, with efficacy repeatedly validated in randomized trials (Gidabayda et al., 2017). The compound retains potent *in-vitro* activity against *Staphylococcus aureus* (Khan et al., 2006), *Haemophilus* influenzae (Gmuender et al., 2001), and Stenotrophomonas maltophilia (Ba et al., 2004). Microbes potentially interacting with ciprofloxacin were ranked by BRMDA-predicted scores. After excluding the 10 drug-microbe pairs already documented in the MDAD database, the 10 highest-ranking organisms were selected for validation. As shown in Table 5, 9 of these 10 predicted associations have been corroborated by independent studies indexed in PubMed.

**TABLE 5:** The top 10 ciprofloxacin associated candidate microbes on MDAD.

Microbe	Evidence
Firmicutes	PMID: 36152132
*Enterococcus* faecium	PMID: 36231256
*Streptococcus* salivarius	PMID: 33083490
Human herpesvirus 5	PMID: 32234012
*Bacteroides* eggerthii	NA
Gardnerella vaginalis	PMID: 8109944
*Escherichia coli*	PMID: 34791074
Eikenella corrodens	PMID: 3530124
*Staphylococcus* capitis	PMID: 10381660
*Serratia marcescens*	PMID: 8031065

*Bacillus* cereus is a Gram-positive, rod-shaped, β-haemolytic bacterium found in soil and food; it is a common cause of food poisoning (Leong et al., 2023). According to relevant literature reports, *Bacillus* cereus is associated with various substances, including copper sulfate (Santo et al., 2011) and silver nitrate (Babu and Gunasekaran, 2009). After removing the three drug-Bacillus cereus pairs already catalogued in the MDAD dataset, 9 of the 10 highest-scoring predictions were corroborated by independent PubMed-indexed publications, as shown in Table 6.

**TABLE 6:** The top 10 *Bacillus* cereus associated candidate drugs on MDAD.

Drug	Evidence
Metronidazole	PMID: 29622912
Cefoxitin	PMID: 2110145
Amoxicillin	PMID: 36444268
Minocycline	PMID: 37154337
Ciprofloxacin	PMID: 40237511
Zidovudine	PMID: 37573457
Resveratrol	PMID: 31367359
Lorazepam	NA
Quercetin	PMID: 16345844
Linezolid	PMID: 35327787

*Mycobacterium tuberculosis*, the aetiologic agent of tuberculosis (Cole et al., 1998), has been experimentally linked to numerous agents, including ciprofloxacin (Shan et al., 2007) and triclosan (Freundlich et al., 2009). After exclusion of the 14 drug-Mycobacterium tuberculosis pairs already documented in the MDAD dataset, 9 of the 10 top-ranked predictions were independently validated by PubMed-indexed studies, as summarised in Table 7.

**TABLE 7:** The top 10 *Mycobacterium tuberculosis* associated candidate drugs on MDAD.

Drug	Evidence
Metronidazole	PMID: 7811018
Cefoxitin	PMID: 23672214
Sulbactam	PMID: 34250791
Amoxicillin	PMID: 26454420
Diazepam	PMID: 10464392
Nafcillin	NA
Itraconazole	PMID: 11248033
Cephalexin	PMID: 33470787
Ticarcillin	PMID: 3105441
Minocycline	PMID: 9145860

It should be noted that the case studies are designed to provide qualitative and translational validation of the proposed model by examining literature support for top-ranked predictions, rather than to serve as a comparative evaluation across different computational methods.

In the case studies, the majority of the top-ranked candidates are supported by existing literature, which qualitatively corroborates that the model prioritizes biologically plausible associations rather than merely reproducing trivial similarities. For entries marked as NA, we conservatively describe them as ‘not yet reported in the queried literature’ (or not indexed in the current search scope), which may reflect incomplete coverage rather than definitive false positives. Overall, these observations are consistent with the model’s rationale of leveraging heterogeneous structure and interaction modeling to prioritize credible candidates.

## Discussion

In the main text, we propose a weighted combination of BAN and RF for predicting microbe-drug associations. Experiments show that this hybrid method can significantly reduce the false positive rate while maintaining high sensitivity. However, every model has its own advantages and disadvantages, which are detailed as follows:

On the advantage side, BAN and RF complement each other in features: BAN automatically captures how microbes, drugs and diseases interact, while RF works well with complex data and redundant information; together, they cut errors of a single model and boost prediction reliability. The method also balances interpretability—BAN’s attention weights can be shown clearly to help guess how things work, and RF ranks key features to let researchers focus on priorities. It is efficient too: related processing reduces BAN’s computing load and avoids prediction biases with small samples, and RF trains fast, enabling efficient checks even on large-scale data.

On the other hand, the model has limitations: it depends on existing data networks—if the data itself is biased, prediction errors will get worse, so future data processing needs improvement; in multi-relationship analysis, BAN often focuses too much on a few interactions and misses potentially important rare ones; RF cannot give continuous prediction results for new, unseen data, which may cause judgment biases sometimes; also, experimental verification is slow-getting accurate microbe-drug interaction data costs a lot, and there are few positive samples, which stops the model from getting better.

## Conclusion

In this study, we present BRMDA, a hybrid framework that couples a bilinear attention network with a random-forest classifier to predict microbe-drug associations. By incorporating disease nodes into the heterogeneous graph and propagating features across all three entity types—microbes, drugs and diseases—BRMDA learns unified embeddings that explicitly capture tri-modal interactions within the BAN module. This design simultaneously boosts robustness and yields biologically interpretable attention weights, offering a transparent and reliable predictor of complex inter-species pharmacological relationships. Nevertheless, the extreme sparsity of microbe–drug associations still carries a tangible risk of over-fitting. Future efforts could bolster robustness by injecting richer biological priors such as metabolic pathways or clinical covariates, compressing the BAN module through parameter-sharing or low-rank factorization, and exploiting graph-based data-augmentation strategies to amplify the scarce training signal. Finally, because MDAD lacks confirmed negatives, pseudo-negatives sampled from unlabeled pairs may contain undiscovered true associations, introducing label noise in evaluation.

## Data Availability

Publicly available datasets were analyzed in this study. This data can be found here: https://github.com/Sun-Yazhou/MDAD.
