# Correction: BRMDA: prediction model for potential microbe-drug associations based on bilinear attention networks and random forest

**DOI:** 10.3389/fgene.2026.1845666

**Published:** 2026-04-21

**Authors:** Ge Yu, Fang Chen, Hui Chen, Shichang Tang, Mingmin Liang, Xianzhi Liu, Bin Zeng, Lei Wang

**Affiliations:** 1 School of Intelligent Equipment, Hunan vocational College of Electronic and Technology, Changsha, China; 2 School of Continuing Education, Central South University of Forestry and Technology, Changsha, China; 3 School of Information Engineering, Hunan vocational College of Electronic and Technology, Changsha, China; 4 Big Data Innovation and Entrepreneurship Education Center of Hunan Province, Changsha University, Changsha, China

**Keywords:** bilinear attention networks, heterogeneous graph, microbe-drug association prediction, multimodal feature fusion, random forest

The **DOI** of the reference for Kuang et al. (2024a) was erroneously written as 10.0.4.162/s12859-024-05687-9. It should be 10.1186/s12859-024-05687-9.

The original article has been updated.

